# Current CML guidelines overemphasize second generation TKIs: revisiting the paradigm

**DOI:** 10.1038/s41408-023-00811-z

**Published:** 2023-03-15

**Authors:** Anushka Walia, Vinay Prasad

**Affiliations:** 1 School of Medicine, University of California, San Francisco, CA USA; 2 Department of Epidemiology and Biostatistics, University of California, San Francisco, CA USA

**Keywords:** Targeted therapies, Chronic myeloid leukaemia

Current National Comprehensive Cancer Network guidelines (NCCN version 1.2023) for chronic-phase chronic myeloid leukemia (CML) recommend second-generation tyrosine kinase inhibitors (2G-TKIs) as first-line therapy for patients with intermediate or high-risk Sokal or Euro scores. In this editorial, we discuss why imatinib should be the preferred first-line drug for all risk groups.

## Risk scores do not accurately predict prognosis

First, CML risk stratification scores are imprecise. It remains unclear whether Sokal scores are associated with CML-related survival in patients receiving TKIs. Analysis of data from the German CML Study IV showed that cumulative incidence probabilities (CIPs) of death due to CML did not differ between low, intermediate, or high-risk groups defined by Sokal scores [1]. 8-year CIPs were found to be 4%, 4%, and 5% for low, intermediate, and high-risk patients, respectively. For Euro scores, high-risk patients had the highest CIPs (12%) but CIPs were lower in the intermediate-risk group than in the low-risk group (2% vs 5%). This suggests that prognostic scores fail to stratify patients by survival outcomes. Outcomes in patients with higher risk scores remain excellent, with a 9-year overall survival (OS) rate of 88% among Sokal non-low-risk patients [2].

The unreliability of Sokal and Euro predictions for patients receiving TKIs is not unexpected. These scores were initially derived based on outcomes of patients receiving chemotherapy or interferon-alpha treatment, and may be less relevant in the TKI area. Sokal and Euro scores show low concordance with each other and the ELTS score (developed for patients receiving imatinib), which better discriminates risk and is currently recommended by European Leukemia Net for baseline risk assessment [2]. The Sokal score is particularly unreliable and is known to over-classify patients as high-risk. Over half of patients classified as high-risk based on Sokal score were found to be non-high risk by ELTS score in one study [2].

## Second-generation-TKIs have no survival benefit but have greater adverse effects

The basis of the NCCN recommendation is that 2G-TKIs lead to improved molecular and cytogenetic responses in CML patients. A meta-analysis of randomized controlled trials comparing second and third-generation TKIs to imatinib showed risk ratios of CCyR (defined as the absence of Ph+ metaphases) and MMR (defined as 3-log reduction in BCR-ABL1 transcripts) at 12 months to be 1.13 and 1.50, respectively [3]. However, it is unclear whether deeper molecular and cytogenic responses translate to improved patient-centered outcomes, and recent evidence suggests the contrary. Bidikian et al. reported long-term outcomes of 131 patients who did not achieve MMR after 2 years of treatment with TKIs, finding that 10-year CML-related OS was 95% if MCyR was achieved and 80% if MCyR was not achieved [4]. MMR is a poor measure of treatment failure, as patients who fail to achieve MMR can still achieve good outcomes. There is very limited data on correlations between CCyR and MMR with OS across multiple randomized controlled trials (i.e. level-1 evidence).

Importantly, 2G-TKIs have failed to demonstrate any improvement in OS or health-related quality of life over imatinib in randomized controlled trials [3]. The ENESTnd study comparing imatinib and nilotinib reported a 10-year OS of 88.3% in the imatinib arm vs 90.3% in the nilotinib (400 mg) arm [5]. No significant difference in OS was found, even though the incidence of progression to accelerated phase/blast phase was suppressed in the nilotinib group. In the DASISION study on imatinib vs dasatinib, 5-year OS was 90.0% and 91.0% in the imatinib and dasatinib arms, respectively [3]. The BFORE trial on imatinib vs bosutinib found similar 12-month OS between treatment groups. While rates of treatment-free remission (TFR) eligibility as defined by molecular measurements by RT-PCR were higher with nilotinib in the ENESTnd study, data on overall TFR success rates was not provided. Actual rates of TFR with imatinib, nilotinib, and dasatinib have been found to be similar (~50%) in discontinuation trials [6]. Based on current data, first-line use of 2G-TKIs provides no real clinical benefit to the patient but adds significant toxicity and cost.

2G-TKIs are arguably more toxic. Specifically, they are associated with cardiovascular, pulmonary, pancreatic, and hepatic toxicities [3]. In the ENESETnd study, 10-year cumulative incidence of cardiovascular events was 24.8% in the nilotinib (300 mg bid) arm as opposed to 6.3% in the imatinib arm [7]. Nilotinib is also associated with glucose tolerance and dyslipidemia, and its use in patients with cardiovascular risk factors or diabetes requires careful consideration. In the DASISION study, dasatinib was found to be a risk factor for pleural effusion and pulmonary hypertension, and patients should be evaluated for pulmonary disease before treatment. The exclusion criteria for trials assessing the efficacy of 2G-TKIs were broader than those used for imatinib alone given their toxicity profiles [8]. Toxicity rates may thus be higher than what trials of 2G-TKIs report. Given that the median age of diagnosis of CML is greater than 60 years, many patients have comorbidities resulting in high risk of treatment-related adverse effects. Patients with comorbidities who are treated with 2G-TKIs require monitoring, resulting in additional medical expenses and time.

2G-TKIs may also be associated with higher likelihood of treatment interruptions. One study using real world data from a claims database found that 59% of patients who received 2G-TKIs had treatment interruptions compared to 45% for imatinib [9]. Similar treatment interruption rates were reported in the ENESTnd and DASISION trials. These studies could not evaluate the reasons for treatment interruption, but toxicity is likely a key factor. Minimizing 2G-TKI treatment interruptions has been shown to lead to better outcomes including greater failure-free survival.

## Pregnancy

Treatment of CML before and during pregnancy requires special consideration, as TKIs are teratogenic and contraindicated during pregnancy. Current guidelines suggest that 2G-TKIs may be preferred in patients assigned female at birth who desire to become pregnant in order to achieve faster molecular responses so that treatment can be safely halted during pregnancy. These recommendations are based on limited observational data suggesting that patients who achieve deep responses prior to conception are more likely to remain in molecular remission if treatment is paused. However, data on long-term effects of losing response during pregnancy is lacking, and the largest analysis of more than 300 pregnancies from the ELN database showed that patients diagnosed with CML during pregnancy or patients with ≤MR3 prior to becoming pregnant still had good outcomes [10]. Interferon remains a safe treatment option during pregnancy that can induce or maintain remission. Moreover, no studies have directly compared outcomes of 2G-TKI and imatinib use prior to pregnancy; such data is necessary to clarify treatment recommendations for younger CML patients who desire to have children.

## Cost

Finally, the much higher cost of 2G-TKIs does not justify any potential benefit as a front-line therapy for CML. Imatinib is the only TKI that is currently off patent, and its price has consequently dropped dramatically in recent years. Generic imatinib costs as low as $4400 per year (average, $35,000/year) without loss of efficacy, while the lowest cost 2G-TKI (nilotinib, Novartis) costs $152,814 per year—a 35-fold difference. Ya-Chen et al. used a decision analytical model to examine the value of 2G-TKIs as opposed to imatinib for frontline therapy in CML from the payer’s perspective [11]. Considering multiple willingness-to-pay thresholds, they reported that the current cost of 2G-TKIs did not justify the higher likelihood of treatment-free remission. Under a very high willingness-to-pay threshold of 200,000/QALY and a 50% difference in 5-year deep molecular response, 2G-TKIs must cost less than $25,000/year to be favorable.

It is important to note that the prices of nilotinib and dasatinib are expected to drop after US patents expire in 2023 and 2025, respectively. However even if cost profiles are similar to imatinib, their use as frontline therapy is not justified on the basis of higher treatment-related toxicities without survival benefit.

## Conclusions

Imatinib should be the preferred first-line drug for chronic phase CML regardless of risk category. Imatinib has a superior toxicity profile than 2G-TKIs and is safer in patients with multiple comorbidities. Currently, in a generic form, imatinib is less than one-thirtieth the cost of the cheapest 2G-TKI. For those who do not respond to imatinib, switching to second-line treatments can still result in good outcomes. The cost and safety benefits of imatinib do not compromise survival, as no differences in OS between imatinib and 2G-TKIs have been established.

## Data Availability

Data sharing is not applicable to this article as no new data were created or analyzed in this study.
